# dia-PASEF Proteomics of Tumor and Stroma LMD Enriched from Archived HNSCC Samples

**DOI:** 10.1021/acsomega.4c11051

**Published:** 2025-03-25

**Authors:** Aswini Panigrahi, Allison L. Hunt, Diego Assis, Matthew Willetts, Bhaskar V. Kallakury, Bruce Davidson, Jaeil Ahn, Thomas P. Conrads, Radoslav Goldman

**Affiliations:** † Department of Oncology, Lombardi Comprehensive Cancer Center, Georgetown University, Washington, District of Columbia 20057, United States; ‡ Women’s Health Integrated Research Center, Women’s Service Line, Inova Health System, Annandale, Virginia 22003, United States; § Bruker Scientific, Billerica, Massachusetts 01821, United States; ∥ Department of Pathology, Lombardi Comprehensive Cancer Center, Georgetown University, Washington, District of Columbia 20007, United States; ⊥ Department of Otolaryngology-Head and Neck Surgery, Medstar Georgetown University Hospital, Washington, District of Columbia 20007, United States; # Department of Biostatistics, Bioinformatics and Biomathematics, Georgetown University, Washington, District of Columbia 20057, United States; ∇ Department of Biochemistry and Molecular & Cellular Biology, Georgetown University, Washington, District of Columbia 20057, United States

## Abstract

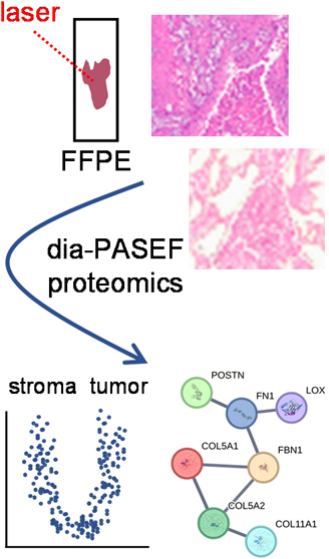

We employed laser microdissection to selectively harvest histology-resolved tumors and stroma from formalin-fixed, paraffin-embedded head and neck squamous cell carcinoma (HNSCC) tissues. Peptide digests from the LMD-enriched HNSCC tissue were analyzed by quantitative mass-spectrometry-based proteomics using a data independent analysis approach. In paired samples, excellent proteome coverage was achieved, having quantified 6668 proteins with a median quantitative coefficient of variation under 10%. Significant differences in relevant functional pathways between the tumor and the stroma regions were observed. Extracellular matrix (ECM) was identified as a major component enriched in the stroma, including many cancer-associated fibroblast signature proteins in this compartment. We demonstrate the potential for comparative deep proteome analysis from a very low starting input in a scalable format. Correlating such results with clinical features or disease progression will likely enable the identification of novel targets for disease classification and interventions.

## Introduction

Head and neck squamous cell carcinoma (HNSCC) is the sixth most common cancer worldwide accounting for approximately 890,000 new cases and 450,000 deaths (4.6% of cancer deaths) annually.^1,2^ HNSCC cells first invade the basement membrane of native epithelium; a large proportion of patients are identified at the time of diagnosis to have lymph node metastasis, which is associated with poor survival.^2−4^ Overall, the response to the available treatments has been moderate. Linking cellular phenotypes to functional proteome states of the HNSCC tumors, including its microenvironment, will add to our understanding of the pathophysiology of the disease and potentially identify drivers of metastasis.

Previously, laser microdissection (LMD) of HNSCC FFPE samples in combination with quantitative mass spectrometry (MS) was introduced by us to characterize normal and tumor lesions.^5^ Recent deep proteomic analysis of tumor and matched normal adjacent tissues, followed by integrated proteogenomic analysis of MS-based proteomic data with genomics and transcriptomics, has identified molecular subtypes with treatment potential.^6^ However, the proteome coverage of early-stage tumors remains insufficient, in part, due to the limited availability of surgical tissues for molecular analysis at this early stage of the disease. Likewise, the spatial resolution of the available HNSCC proteomic data and its correlation with clinicopathological features are very limited. Recent advances in the LMD technology and MS-based techniques allow deep proteome analysis from very low input peptide samples.^7−12^

Motivated by these advancements, in this study, we selectively harvested tumor and stroma regions from HNSCC FFPE samples using LMD for independent MS-based proteomics analysis using a data independent analysis (DIA) approach. We achieved deep proteome coverage, identified significant differences in functional pathways of tumor and stroma, and demonstrated the potential for comparative deep proteome analysis in a scalable format.

## Results and Discussion

### dia-PASEF LC-MS Analysis of Tumor and Stroma

**Figure 1:**
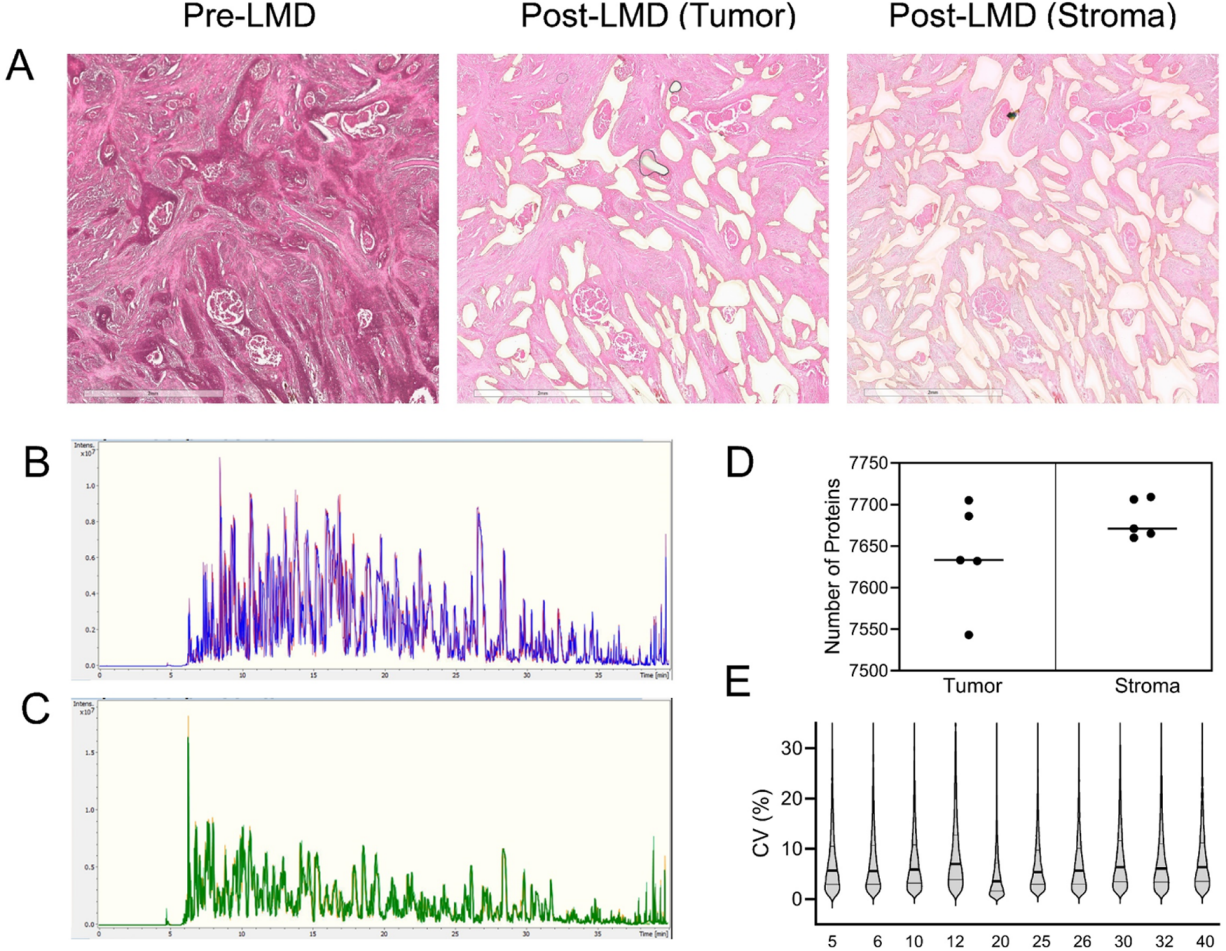
Representative (A) H&E stained FFPE tissue sections on PEN membrane slides before (left) and after LMD harvest of tumor (middle) and stroma (right). Base peak chromatograms showing the overlay of triplicate runs of an enriched (B) tumor and (C) paired stroma sample. (D) Graphical representation of the number of proteins identified with 2 or more peptide matches in the enriched tumor and stroma samples. The median is indicated by a line. (E) Schematic representation of the CV value spread of quantitation for each sample; median (bold line) and upper and lower quartiles (thin line) are indicated.

Tumor and stroma were independently harvested by LMD from five HNSCC patient FFPE specimens for analysis by DIA MS (Figure 1A). We obtained an average of 0.97 μg of tryptic peptides per mm^2^ of tumor and 0.48 μg/mm^2^ of stroma. The samples were analyzed in triplicate (500 ng of peptide digest per injection) on a timsTOF HT MS (40 min run time, equating to an ∼2 h MS analytical time per sample). Excellent chromatographic reproducibility was observed over the entire sample batch run (Figure 1B,C). A total of 8759 protein groups were identified from 98,005 peptide matches, among which 8498 proteins were from LMD-enriched tumor epithelium and 8396 proteins from the stromal region, with 94% of proteins coquantified in both tumor and stroma. Of these proteins, 7825 were identified with two or more peptide matches in at least one sample (Supporting Table S1A). We observed similar protein coverage (range 7543–7709) in each sample (Figure 1D). Across all patient samples, 6668 proteins were coquantified with the median coefficients of variation (CVs) consistently under 10% (Figure 1E).

### Differential Proteome in Tumor and Stroma

**Figure 2:**
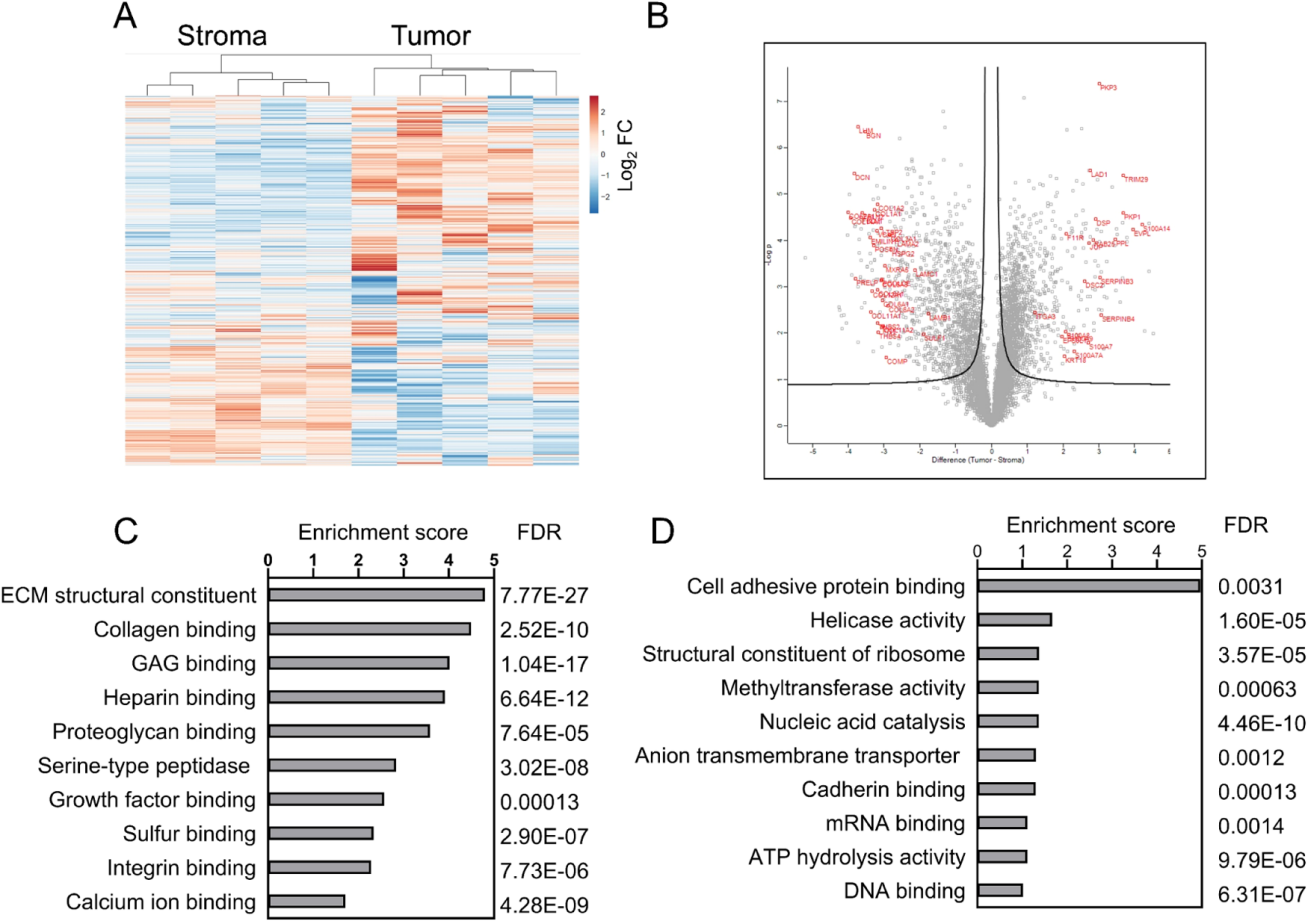
Functional association of upregulated proteins. (A) Unsupervised hierarchical cluster analysis of stroma and tumor region proteins based on abundance. (B) Volcano plot of protein abundance. In volcano plot, the box dot plots of significantly changed proteins between the regions are indicated, and selected candidates are in red. Selected enriched functional pathways (String analysis) in stroma (C) and tumor (D) are shown with enrichment score and false discovery rate (FDR).

Unsupervised hierarchical cluster analysis of the proteins quantified revealed that the stroma and tumor samples clustered independently (Figure 2A). Differential analysis identified 2655 significantly altered proteins between tumor epithelium and stroma (Figure 2B and Supporting Table S1B). Gene ontology molecular functional pathway enrichment analysis of these proteins (Supporting Table 2) identified several functional groups relevant to the stromal and tumor regions (Figure 2C,D). Notably, extracellular matrix (ECM) structural constituent (GO:0005201), collagen binding (GO:0005518), glycosaminoglycan binding (GO:0005539), and heparin binding (GO:0008201) were enriched in the stroma. Comparatively, nucleic acid catalytic activity, DNA binding, ATP hydrolysis, and helicase and ribosome constituents were enriched in tumor epithelium. In the top 100 tumor-enriched proteins, we identified candidates with functional and physical association with cell–cell adhesion (DSC2, DSP, JUP, KRT18, PKP3, and TRIM29), cadherin binding (EPS8L1, EVPL, F11R, JUP, KRT18, LAD1, PKP1, PKP3, PPL, TRIM29), and calcium-dependent protein binding (S100A14, S100A7, S100A7A, S100A8, S100A9). These proteins are highlighted in the volcano plot (Figure 2B, right side).

### Extracellular Matrix

The extracellular matrix (ECM) is composed of structural proteins such as laminins, collagens, proteoglycans, and fibronectin.^13,14^ Several components of ECM structural constituent, including BGN, COL11A1, COL11A2, COL12A1, COL14A1, COL1A1, COL1A2, COL2A1, COL3A1, COL6A1, COL6A2, COL6A3, COL8A1, COMP, DCN, EMILIN1, FBLN1, FBLN2, FN1, HSPG2, LUM, MXRA5, PRELP, and VCAN, were among the top 100 most abundant proteins in stroma (Figure 2C and Supporting Tables 1B and 2), and these are highlighted in the volcano plot (Figure 2B, left side). Several of these stromal-enriched proteins (e.g., COL11A1, COMP, FN1, POSTN, SULF1, and THBS2) are signature markers of COL11A1-expressing cancer-associated fibroblasts (CAFs)^15^ and are detectable in several cancers, including HNSCC.^16^ COL11A1 CAFs are a common driver of aggressive cancer behavior,^15^ and human sulfatase 1 (SULF1)-positive CAFs were recently demonstrated to promote colorectal cancer development.^17^

CAFs are key component of the tumor microenvironment due to their ability to synthesize and remodel the ECM and perform diverse functions.^18−20^ The CAF-mediated ECM remodeling includes deposition of collagen, synthesis of proteoglycans, and overexpression of remodeling enzymes, e.g., matrix metalloproteinases (MMPs).^21,22^ We observed that collagens (listed above), proteoglycans, and MMPs (specifically, MMP2, 3, 10, and 14) were more abundant in stroma. The CAF-mediated ECM remodeling is dynamic, providing signals that regulate the tumor cells and their interaction with the microenvironment and impacts their migration and metastatic potential.^23^ Characterization of the stromal microenvironment is valuable,^18,24,25^ and the pathology-guided LMD enrichment method coupled with the high sensitivity of liquid chromatography with tandem mass spectrometry (LC-MS/MS) in HNSCC tissues will provide clues connecting the disease pathology to outcomes.

One limitation of this study is a small sample set, which limits our ability to perform rigorous informatic analyses of specific disease association. Nonetheless, KEGG pathway enrichment and protein–protein interaction prediction of ECM proteins were performed to obtain snapshot characterizations of biological processes and protein components within the HNSCC tumor microenvironment. Table 1 lists the proteins that are associated with the ECM–receptor interaction, protein digestion and absorption, signaling, and pathways in cancer.

**Table 1:** KEGG Pathway Enrichment of ECM Proteins That Changed Significantly between Stromal and Tumor Regions

KEGG ID	description	gene count	strength	FDR	matching proteins
hsa04512	ECM–receptor interaction	26/88	1.56	3.89 × 10^–28^	ITGB4, COMP, LAMB1, COL1A1, LAMA4, LAMC1, THBS1, VWF, TNC, COL6A3, COL1A2, COL6A2, LAMB2, THBS4, FN1, COL4A2, COL6A1, THBS2, THBS3, HSPG2, COL4A1, COL2A1, LAMA1, LAMA2, TNXB, DAG1
hsa04974	protein digestion and absorption	23/100	1.45	7.81 × 10^–23^	COL1A1, COL21A1, COL8A1, COL5A3, COL6A3, CPA3, COL1A2, COL14A1, COL6A2, COL3A1, COL12A1, ELN, COL18A1, COL4A2, COL6A1, COL11A1, COL5A1, COL16A1, COL5A2, COL15A1, COL4A1, COL2A1, COL28A1
hsa04610	complement and coagulation cascades	16/82	1.38	8.09 × 10^–15^	F9, F12, VWF, F13A1, SERPING1, FGB, F2, C1QB, A2M, FGG, SERPINC1, C1QC, C1QA, SERPINA1, KNG1, FGA
hsa04510	focal adhesion	24/195	1.18	2.47 × 10^–18^	ITGB4, COMP, LAMB1, COL1A1, LAMA4, LAMC1, THBS1, VWF, TNC, COL6A3, COL1A2, COL6A2, LAMB2, THBS4, FN1, COL4A2, COL6A1, THBS2, THBS3, COL4A1, COL2A1, LAMA1, LAMA2, TNXB
hsa05222	small cell lung cancer	9/92	1.08	5.91 × 10^–06^	LAMB1, LAMA4, LAMC1, LAMB2, FN1, COL4A2, COL4A1, LAMA1, LAMA2
hsa04151	PI3K-Akt signaling pathway	24/349	0.92	2.69 × 10^–13^	ITGB4, COMP, LAMB1, COL1A1, LAMA4, LAMC1, THBS1, VWF, TNC, COL6A3, COL1A2, COL6A2, LAMB2, THBS4, FN1, COL4A2, COL6A1, THBS2, THBS3, COL4A1, COL2A1, LAMA1, LAMA2, TNXB
hsa04611	platelet activation	8/122	0.9	0.00043	COL1A1, VWF, COL1A2, COL3A1, FGB, F2, FGG, FGA
hsa04350	TGF-β signaling pathway	5/91	0.82	0.024	DCN, THBS1, FBN1, FMOD, LTBP1
hsa04926	relaxin signaling pathway	6/126	0.76	0.0181	MMP2, COL1A1, COL1A2, COL3A1, COL4A2, COL4A1
hsa05205	proteoglycans in cancer	8/194	0.7	0.0072	DCN, MMP2, COL1A1, THBS1, LUM, COL1A2, FN1, HSPG2
hsa05200	pathways in cancer	12/515	0.45	0.0261	MMP2, LAMB1, LAMA4, LAMC1, LAMB2, F2, FN1, COL4A2, COL4A1, LAMA1, LAMA2, KNG1

**Figure 3:**
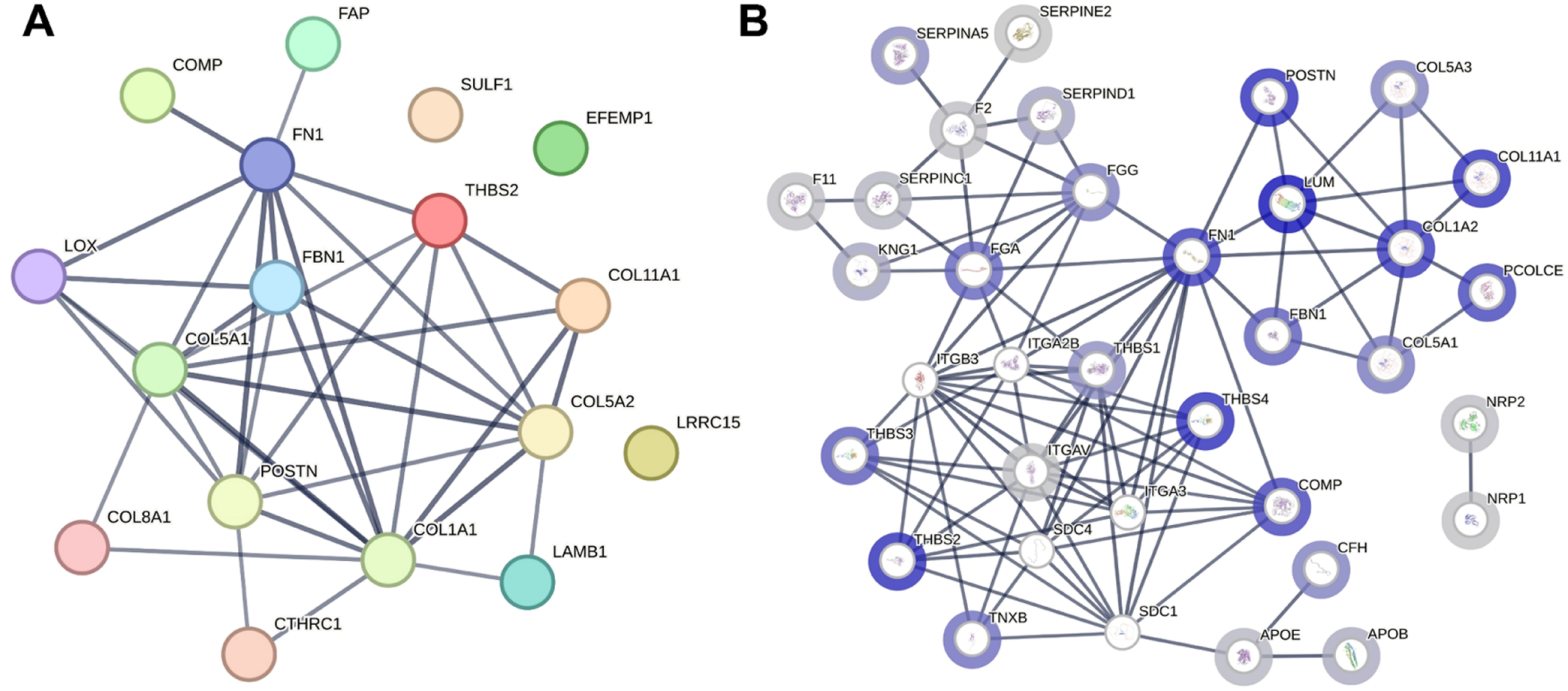
Protein–protein association map of stromal compartment upregulated (A) CAF markers and (B) heparin binding proteins.

A predicted protein–protein association map (deduced from the STRING database analysis) of the stromal CAF-associated proteins is presented in Figure 3A. Many biological processes are mediated by the protein–protein interaction between stromal CAFs and other tumor-associated cells. One of the CAF proteins, SULF1, edits 6-*O*-sulfation of heparan sulfate proteoglycans (HSPGs).^26^ This affects the interaction of HSPGs with ligands and signaling molecules, e.g., fibronectin, Wnts, TGF-β1, and FGFs.^27−29^ In an earlier study, we reported that SULF1 RNA expression is significantly higher in fibroblasts compared with that in tumor epithelial cells and has strong correlation with the expression of CAF1 marker genes.^16^ We have shown that SULF1 RNA and protein are elevated in multiple cancer tissues and that increased expression is associated with poor survival outcomes.^16^ A recent study demonstrated that SULF1+ CAFs portend the poor survival of colorectal cancer patients.^17^

In this study, we observed the enrichment of SULF1 in the LMD-enriched stromal microenvironment. In addition to SULF1, a protein with high affinity for heparin/heparan sulfate,^30^ other heparin binding proteins were significantly enriched in the stroma, including many CAF-associated proteins, e.g., COL11A1, COMP, FN1, and POSTN. STRING analysis of the heparin binding proteins (Figure 3B) revealed that a large majority of the stromal-enriched heparin binding proteins are predicted to have functional and physical association, suggesting that there is likely a heparan sulfate proteoglycan interacting network that mediates functional signaling in the tumor microenvironment (TME).

Overall, we demonstrate excellent proteome coverage of the HNSCC tumor and stromal microenvironment from LMD-enriched tissue samples using a highly sensitive and quantitative dia-PASEF LC-MS/MS workflow. Though our sample set was limited, we quantified proteomic alterations between the HNSCC stroma and tumor using a minimal 1.5 μg of isolated tryptic peptides for triplicate analysis per tissue-specific region. The methodology allows comparative deep proteome analysis in a scalable format and allows the connection of the enriched proteomes to pathologic features of the tumor tissues. While the peptide yield from the stromal regions was lower, the sensitivity of the LC-MS/MS workflow allows reproducible proteome analysis with coverage comparable to that of the enriched tumor regions. Future expansion of this study in a large sample cohort using this workflow will allow for further identification and validation of proteins relevant to disease progression and survival.

## Materials and Methods

### Experimental Design

In this study, proteomic profiling of five patient samples was conducted. From each tissue specimen, the tumor and stroma-enriched regions were independently harvested by LMD. LC-MS analysis of these paired samples was performed in triplicate to demonstrate the technical reproducibility. Statistical analysis was performed to determine the coefficient of variation (CV) for each sample analysis. The relative protein difference between the two paired sample groups, i.e., enriched tumor (*n* = 5) and enriched stroma (*n* = 5), were compared, and Student’s *t* test was used to compute *p*-values.

### Patient Specimens

Study participants were enrolled between 1995 and 2020 at the Department of Otolaryngology-Head and Neck Surgery, MedStar Georgetown University Hospital, under a protocol approved by the MedStar Health Research Institute-Georgetown University Oncology Institutional Review Board. Thin-sectioned formalin-fixed paraffin-embedded (FFPE) HNSCC tumor specimens mounted on poly(ethylene naphthalate) (PEN) membrane slides were prepared by Histology & Tissue Shared Resource at Georgetown University Medical Center. The tissue slides were stained with hematoxylin and eosin (H&E), imaged by microscopy, and reviewed by a board-certified pathologist for annotation of the tumor and stroma areas to inform LMD harvest.

### Laser Microdissection

An average of 42 mm^2^ (range 13–54 mm^2^) and 32 mm^2^ (range 15–50 mm^2^) of tumor epithelium and stroma, respectively, was harvested by LMD as recently described.^7^ Representative micrographs before and after LMD harvest were imaged using an Aperio AT2 scanner (Leica Microsystems, Wetzlar, Germany). The LMD-enriched samples were digested using trypsin and pressure cycling technology (PCT), and the peptide samples were desalted using C18 cartridges.

### LC-MS Analysis

A timsTOF HT mass spectrometer connected to a nanoElute 2 LC system via a CaptiveSpray 2 source (Bruker, Billerica, MA) was used for dia-PASEF LC-MS/MS analysis. Each sample was analyzed in triplicate (except one sample in duplicate, which was used for the initial method optimization and availability was limited). The peptides were separated by a C18 IonOpticks column (particle size 1.6 μm, 75 μM μm ID, 25 cm length) at a flow rate of 0.25 μL/min; solvent A (0.1% formic acid in water), solvent B (0.1% formic acid in acetonitrile), gradient of 0–28 min 5–23% B, 28–32 min to 30% B, 32–36 min to 90% B, and hold at 90% B to 40 min. For dia-PASEF analysis, the window scheme was calculated using the py_diAID tool (https://github.com/MannLabs/pydiaid). The capillary voltage was set at 1600 V, stepping collision energy at 32, 40, and 50 eV, dia-PASEF scan range 100–1700 *m*/*z* in positive mode, and IMS service ramp time of 100 ms.

### Data Analysis

Acquired mass spectrometry data was searched against Uniprot-Human-reviewed database (20,383 protein entries) using the directDIA+ workflow (Spectronaut 18 software) for protein identification and quantification using BGS default settings. Unsupervised hierarchical clustering analyses were done using Clustvis (v 1.2.0) in R package (v 3.6.2).^31^ Perseus software (v 2.0.11) was used to compare the relative abundance of each protein in two sample groups via performing Student’s *t* tests, log-fold changes, and Volcano plot analysis.^32^ All raw *p*-values were adjusted using the false discovery rate (FDR). The Gene Ontology (GO) molecular functional pathway enrichment analysis was performed using the STRING database (string-db.org, v 12.0). The proteins with statistically significant abundance variations between the two groups were uploaded along with the values for the analysis. KEGG (Kyoto Encyclopedia of Genes and Genomes) pathway enrichment of specific protein groups was further extracted from the STRING output. The protein–protein interaction map was generated from the STRING functional and physical protein association network analysis. GraphPad Prism software (version 10.2.3) was used for data visualization.

## Data Availability

The mass spectrometry data have been deposited to the jPOST repository.^33^ The accession numbers are PXD054650 for ProteomeXchange and JPST003262 for jPOST (https://repository.jpostdb.org/preview/140548518366b29246602a8 Access key: 1101).

## Supporting Information Available

Table S1: (A) List of proteins identified with two or more peptide matches in at least one of the tumors or stroma-enriched sample, and the quantitative value in each sample is presented; (B) relative abundance of proteins that changed significantly between paired tumor and stroma groups; Log_2_ fold change and *p*-value for each protein are presented (XLSX)
Table S2: Gene ontology (GO) functional enrichment analysis of the proteins that changed significantly between stroma and tumor region; matched proteins to the molecular functional pathway, enrichment score, and FDR is presented (XLSX)

## References

[ref1] Barsouk A.; Aluru J. S.; Rawla P.; Saginala K.; Barsouk A. Epidemiology, Risk Factors, and Prevention of Head and Neck Squamous Cell Carcinoma. Med. Sci. 2023, 11 (2), 42 10.3390/medsci11020042. PMC10304137 37367741

[ref2] Johnson D. E.; Burtness B.; Leemans C. R.; Lui V. W. Y.; Bauman J. E.; Grandis J. R. Head and Neck Squamous Cell Carcinoma. Nat. Rev. Dis Primers 2020, 6 (1), 92 10.1038/s41572-020-00224-3. 33243986 PMC7944998

[ref3] Sanderson R. J. Squamous Cell Carcinomas of the Head and Neck. BMJ 2002, 325, 822 10.1136/bmj.325.7368.822. 12376446 PMC1124330

[ref4] Leusink F. K.; Koudounarakis E.; Frank M. H.; Koole R.; van Diest P. J.; Willems S. M. Cathepsin K Associates with Lymph Node Metastasis and Poor Prognosis in Oral Squamous Cell Carcinoma. BMC Cancer 2018, 18 (1), 385 10.1186/s12885-018-4315-8. 29618339 PMC5885370

[ref5] Patel V.; Hood B. L.; Molinolo A. A.; Lee N. H.; Conrads T. P.; Braisted J. C.; Krizman D. B.; Veenstra T. D.; Gutkind J. S. Proteomic Analysis of Laser-Captured Paraffin-Embedded Tissues: A Molecular Portrait of Head and Neck Cancer Progression. Clin. Cancer Res. 2008, 14 (4), 1002–1014. 10.1158/1078-0432.CCR-07-1497. 18281532

[ref6] Huang C.; Chen L.; Savage S. R.; Eguez R. V.; Dou Y.; Li Y.; da Veiga Leprevost F.; Jaehnig E. J.; Lei J. T.; Wen B.; Schnaubelt M.; Krug K.; Song X.; Cieślik M.; Chang H.-Y.; Wyczalkowski M. A.; Li K.; Colaprico A.; Li Q. K.; Clark D. J.; Hu Y.; Cao L.; Pan J.; Wang Y.; Cho K.-C.; Shi Z.; Liao Y.; Jiang W.; Anurag M.; Ji J.; Yoo S.; Zhou D. C.; Liang W.-W.; Wendl M.; Vats P.; Carr S. A.; Mani D. R.; Zhang Z.; Qian J.; Chen X. S.; Pico A. R.; Wang P.; Chinnaiyan A. M.; Ketchum K. A.; Kinsinger C. R.; Robles A. I.; An E.; Hiltke T.; Mesri M.; Thiagarajan M.; Weaver A. M.; Sikora A. G.; Lubiński J.; Wierzbicka M.; Wiznerowicz M.; Satpathy S.; Gillette M. A.; Miles G.; Ellis M. J.; Omenn G. S.; Rodriguez H.; Boja E. S.; Dhanasekaran S. M.; Ding L.; Nesvizhskii A. I.; El-Naggar A. K.; Chan D. W.; Zhang H.; Zhang B. Clinical Proteomic Tumor Analysis Consortium. Proteogenomic Insights into the Biology and Treatment of HPV-Negative Head and Neck Squamous Cell Carcinoma. Cancer Cell 2021, 39 (3), 361–379. 10.1016/j.ccell.2020.12.007. 33417831 PMC7946781

[ref7] Hunt A. L.; Bateman N. W.; Barakat W.; Makohon-Moore S.; Hood B. L.; Conrads K. A.; Zhou M.; Calvert V.; Pierobon M.; Loffredo J.; Litzi T. J.; Oliver J.; Mitchell D.; Gist G.; Rojas C.; Blanton B.; Robinson E. L.; Odunsi K.; Sood A. K.; Casablanca Y.; Darcy K. M.; Shriver C. D.; Petricoin E. F.; Rao U. N. M.; Maxwell G. L.; Conrads T. P. Extensive Three-Dimensional Intratumor Proteomic Heterogeneity Revealed by Multiregion Sampling in High-Grade Serous Ovarian Tumor Specimens. iScience 2021, 24 (7), 102757 10.1016/j.isci.2021.102757. 34278265 PMC8264160

[ref8] Mitchell D.; Hunt A. L.; Conrads K. A.; Hood B. L.; Makohon-Moore S. C.; Rojas C.; Maxwell G. L.; Bateman N. W.; Conrads T. P. Industrialized, Artificial Intelligence-Guided Laser Microdissection for Microscaled Proteomic Analysis of the Tumor Microenvironment. J. Visualized Exp. 2022, 184, e64171 10.3791/64171. 35723500

[ref9] Truong J. X. M.; Rao S. R.; Ryan F. J.; Lynn D. J.; Snel M. F.; Butler L. M.; Trim P. J. Spatial MS Multiomics on Clinical Prostate Cancer Tissues. Anal. Bioanal. Chem. 2024, 416 (7), 1745–1757. 10.1007/s00216-024-05178-z. 38324070

[ref10] Mund A.; Coscia F.; Kriston A.; Hollandi R.; Kovács F.; Brunner A.-D.; Migh E.; Schweizer L.; Santos A.; Bzorek M.; Naimy S.; Rahbek-Gjerdrum L. M.; Dyring-Andersen B.; Bulkescher J.; Lukas C.; Eckert M. A.; Lengyel E.; Gnann C.; Lundberg E.; Horvath P.; Mann M. Deep Visual Proteomics Defines Single-Cell Identity and Heterogeneity. Nat. Biotechnol. 2022, 40 (8), 1231–1240. 10.1038/s41587-022-01302-5. 35590073 PMC9371970

[ref11] Meier F.; Brunner A.-D.; Frank M.; Ha A.; Bludau I.; Voytik E.; Kaspar-Schoenefeld S.; Lubeck M.; Raether O.; Bache N.; Aebersold R.; Collins B. C.; Röst H. L.; Mann M. diaPASEF: Parallel Accumulation-Serial Fragmentation Combined with Data-Independent Acquisition. Nat. Methods 2020, 17 (12), 1229–1236. 10.1038/s41592-020-00998-0. 33257825

[ref12] Demichev V.; Szyrwiel L.; Yu F.; Teo G. C.; Rosenberger G.; Niewienda A.; Ludwig D.; Decker J.; Kaspar-Schoenefeld S.; Lilley K. S.; Mülleder M.; Nesvizhskii A. I.; Ralser M. Dia-PASEF Data Analysis Using FragPipe and DIA-NN for Deep Proteomics of Low Sample Amounts. Nat. Commun. 2022, 13 (1), 3944 10.1038/s41467-022-31492-0. 35803928 PMC9270362

[ref13] Wenta T.; Nastaly P.; Lipinska B.; Manninen A. Remodeling of the Extracellular Matrix by Serine Proteases as a Prerequisite for Cancer Initiation and Progression. Matrix Biol. 2024, 134, 197–219. 10.1016/j.matbio.2024.10.007. 39500383

[ref14] Sleeboom J. J. F.; van Tienderen G. S.; Schenke-Layland K.; van der Laan L. J. W.; Khalil A. A.; Verstegen M. M. A. The Extracellular Matrix as Hallmark of Cancer and Metastasis: From Biomechanics to Therapeutic Targets. Sci. Transl Med. 2024, 16 (728), eadg3840 10.1126/scitranslmed.adg3840. 38170791

[ref15] Zhu K.; Cai L.; Cui C.; de Los Toyos J. R.; Anastassiou D. Single-Cell Analysis Reveals the Pan-Cancer Invasiveness-Associated Transition of Adipose-Derived Stromal Cells into COL11A1-Expressing Cancer-Associated Fibroblasts. PLoS Comput. Biol. 2021, 17 (7), e1009228 10.1371/journal.pcbi.1009228. 34283835 PMC8323949

[ref16] Yang Y.; Ahn J.; Edwards N. J.; Benicky J.; Rozeboom A. M.; Davidson B.; Karamboulas C.; Nixon K. C. J.; Ailles L.; Goldman R. Extracellular Heparan 6-O-Endosulfatases SULF1 and SULF2 in Head and Neck Squamous Cell Carcinoma and Other Malignancies. Cancers 2022, 14 (22), 5553 10.3390/cancers14225553. 36428645 PMC9688903

[ref17] Wang H.; Chen J.; Chen X.; Liu Y.; Wang J.; Meng Q.; Wang H.; He Y.; Song Y.; Li J.; Ju Z.; Xiao P.; Qian J.; Song Z. Cancer-Associated Fibroblasts Expressing Sulfatase 1 Facilitate VEGFA-Dependent Microenvironmental Remodeling to Support Colorectal Cancer. Cancer Res. 2024, 84 (20), 3371–3387. 10.1158/0008-5472.CAN-23-3987. 39250301

[ref18] Li X.; González-Maroto C.; Tavassoli M. Crosstalk between CAFs and Tumour Cells in Head and Neck Cancer. Cell Death Discovery 2024, 10 (1), 303 10.1038/s41420-024-02053-9. 38926351 PMC11208506

[ref19] de Visser K. E.; Joyce J. A. The Evolving Tumor Microenvironment: From Cancer Initiation to Metastatic Outgrowth. Cancer Cell 2023, 41 (3), 374–403. 10.1016/j.ccell.2023.02.016. 36917948

[ref20] Sahai E.; Astsaturov I.; Cukierman E.; DeNardo D. G.; Egeblad M.; Evans R. M.; Fearon D.; Greten F. R.; Hingorani S. R.; Hunter T.; Hynes R. O.; Jain R. K.; Janowitz T.; Jorgensen C.; Kimmelman A. C.; Kolonin M. G.; Maki R. G.; Powers R. S.; Puré E.; Ramirez D. C.; Scherz-Shouval R.; Sherman M. H.; Stewart S.; Tlsty T. D.; Tuveson D. A.; Watt F. M.; Weaver V.; Weeraratna A. T.; Werb Z. A Framework for Advancing Our Understanding of Cancer-Associated Fibroblasts. Nat. Rev. Cancer 2020, 20 (3), 174–186. 10.1038/s41568-019-0238-1. 31980749 PMC7046529

[ref21] Sangaletti S.; Chiodoni C.; Tripodo C.; Colombo M. P. The Good and Bad of Targeting Cancer-Associated Extracellular Matrix. Curr. Opin Pharmacol. 2017, 35, 75–82. 10.1016/j.coph.2017.06.003. 28734136

[ref22] Yuan Z.; Li Y.; Zhang S.; Wang X.; Dou H.; Yu X.; Zhang Z.; Yang S.; Xiao M. Extracellular Matrix Remodeling in Tumor Progression and Immune Escape: From Mechanisms to Treatments. Mol. Cancer 2023, 22 (1), 48 10.1186/s12943-023-01744-8. 36906534 PMC10007858

[ref23] Kai F.; Drain A. P.; Weaver V. M. The Extracellular Matrix Modulates the Metastatic Journey. Dev. Cell 2019, 49 (3), 332–346. 10.1016/j.devcel.2019.03.026. 31063753 PMC6527347

[ref24] Mukherjee P.; Zhou X.; Benicky J.; Panigrahi A.; Aljuhani R.; Liu J.; Ailles L.; Pomin V. H.; Wang Z.; Goldman R. Heparan-6-O-Endosulfatase 2 Promotes Invasiveness of Head and Neck Squamous Carcinoma Cell Lines in Co-Cultures with Cancer-Associated Fibroblasts. Cancers 2023, 15 (21), 5168 10.3390/cancers15215168. 37958342 PMC10650326

[ref25] Principe S.; Mejia-Guerrero S.; Ignatchenko V.; Sinha A.; Ignatchenko A.; Shi W.; Pereira K.; Su S.; Huang S. H.; O’Sullivan B.; Xu W.; Goldstein D. P.; Weinreb I.; Ailles L.; Liu F.-F.; Kislinger T. Proteomic Analysis of Cancer-Associated Fibroblasts Reveals a Paracrine Role for MFAP5 in Human Oral Tongue Squamous Cell Carcinoma. J. Proteome Res. 2018, 17 (6), 2045–2059. 10.1021/acs.jproteome.7b00925. 29681158

[ref26] Morimoto-Tomita M.; Uchimura K.; Werb Z.; Hemmerich S.; Rosen S. D. Cloning and Characterization of Two Extracellular Heparin-Degrading Endosulfatases in Mice and Humans. J. Biol. Chem. 2002, 277 (51), 49175–49185. 10.1074/jbc.M205131200. 12368295 PMC2779716

[ref27] Fellgett S. W.; Maguire R. J.; Pownall M. E. Sulf1 Has Ligand-Dependent Effects on Canonical and Non-Canonical Wnt Signalling. J. Cell Sci. 2015, 128 (7), 1408–1421. 10.1242/jcs.164467. 25681501 PMC4379729

[ref28] Uchimura K.; Morimoto-Tomita M.; Bistrup A.; Li J.; Lyon M.; Gallagher J.; Werb Z.; Rosen S. D. HSulf-2, an Extracellular Endoglucosamine-6-Sulfatase, Selectively Mobilizes Heparin-Bound Growth Factors and Chemokines: Effects on VEGF, FGF-1, and SDF-1. BMC Biochem. 2006, 7, 2 10.1186/1471-2091-7-2. 16417632 PMC1386684

[ref29] Pye D. A.; Vivès R. R.; Hyde P.; Gallagher J. T. Regulation of FGF-1 Mitogenic Activity by Heparan Sulfate Oligosaccharides Is Dependent on Specific Structural Features: Differential Requirements for the Modulation of FGF-1 and FGF-2. Glycobiology 2000, 10 (11), 1183–1192. 10.1093/glycob/10.11.1183. 11087710

[ref30] Frese M.-A.; Milz F.; Dick M.; Lamanna W. C.; Dierks T. Characterization of the Human Sulfatase Sulf1 and Its High Affinity Heparin/Heparan Sulfate Interaction Domain. J. Biol. Chem. 2009, 284 (41), 28033–28044. 10.1074/jbc.M109.035808. 19666466 PMC2788855

[ref31] Metsalu T.; Vilo J. ClustVis: A Web Tool for Visualizing Clustering of Multivariate Data Using Principal Component Analysis and Heatmap. Nucleic Acids Res. 2015, 43 (W1), W566–W570. 10.1093/nar/gkv468. 25969447 PMC4489295

[ref32] Tyanova S.; Temu T.; Sinitcyn P.; Carlson A.; Hein M. Y.; Geiger T.; Mann M.; Cox J. The Perseus Computational Platform for Comprehensive Analysis of (Prote)Omics Data. Nat. Methods 2016, 13 (9), 731–740. 10.1038/nmeth.3901. 27348712

[ref33] Okuda S.; Watanabe Y.; Moriya Y.; Kawano S.; Yamamoto T.; Matsumoto M.; Takami T.; Kobayashi D.; Araki N.; Yoshizawa A. C.; Tabata T.; Sugiyama N.; Goto S.; Ishihama Y. jPOSTrepo: An International Standard Data Repository for Proteomes. Nucleic Acids Res. 2017, 45 (D1), D1107–D1111. 10.1093/nar/gkw1080. 27899654 PMC5210561

